# A rapid virus-induced gene silencing (VIGS) method for assessing resistance and susceptibility to cassava mosaic disease

**DOI:** 10.1186/s12985-017-0716-6

**Published:** 2017-03-07

**Authors:** Getu Beyene, Raj Deepika Chauhan, Nigel J. Taylor

**Affiliations:** Donald Danforth Plant Science Center, 975 North Warson Road, St. Louis, MO 63132 USA

**Keywords:** Cassava mosaic disease, VIGS, SPINDLY, Resistance, Susceptible, Geminivirus

## Abstract

**Background:**

Cassava mosaic disease (CMD) is a major constraint to cassava production in sub-Saharan Africa. Under field conditions, evaluation for resistance to CMD takes 12–18 months, often conducted across multiple years and locations under pressure from whitefly-mediated transmission. Under greenhouse or laboratory settings, evaluation for resistance or susceptibility to CMD involves transmission of the causal viruses from an infected source to healthy plants through grafting, or by using *Agrobacterium*-mediated or biolistic delivery of infectious clones. Following inoculation, visual assessment for CMD symptom development and recovery requires 12–22 weeks. Here we report a rapid screening system for determining resistance and susceptibility to CMD based on virus-induced gene silencing (VIGS) of an endogenous cassava gene.

**Results:**

A VIGS vector was developed based on an infectious clone of the virulent strain of *East African cassava mosaic virus* (EACMV-K201). A sequence from the cassava (*Manihot esculenta*) ortholog of Arabidopsis *SPINDLY* (*SPY*) was cloned into the CP position of the DNA-A genomic component and used to inoculate cassava plants by Helios® Gene Gun microparticle bombardment. Silencing of *Manihot esculenta* SPY (*MeSPY*) using MeSPY1-VIGS resulted in shoot-tip necrosis followed by death of the whole plant in CMD susceptible cassava plants within 2–4 weeks. CMD resistant cultivars were not affected and remained healthy after challenge with MeSPY1-VIGS. Significantly higher virus titers were detected in CMD-susceptible cassava lines compared to resistant controls and were correlated with a concomitant reduction in *MeSPY* expression in susceptible plants.

**Conclusions:**

A rapid VIGS-based screening system was developed for assessing resistance and susceptibility to CMD. The method is space and resource efficient, reducing the time required to perform CMD screening to as little as 2–4 weeks. It can be employed as a high throughput rapid screening system to assess new cassava cultivars and for screening transgenic, gene edited and breeding lines under controlled growth conditions.

**Electronic supplementary material:**

The online version of this article (doi:10.1186/s12985-017-0716-6) contains supplementary material, which is available to authorized users.

## Background

The starchy storage root of cassava serves as a staple food for millions of people in Africa. In 2014, over 50% of the world’s cassava production took place in sub-Saharan Africa, where 146.8 million tons were harvested [1]. While cassava is resilient to abiotic stresses such as prolonged drought [2], its production is constrained by the two viral diseases, cassava mosaic disease (CMD) and cassava brown streak disease (CBSD) [3]. Cassava mosaic disease is caused by a cassava mosaic geminivirus (CMG) complex. CMGs are single-stranded bipartite DNA viruses in the family Geminiviridae, genus *Begomoviru*s, comprised of 11 species, two of which are present in the Indian sub-continent, with the rest endemic to Africa [4, 5].

Improvement programs for development of CMD-resistant cassava germplasm include introgression of polygenic recessive resistance from the related species *Manihot glaziovii* (CMD1), identification of monogenic dominant resistance in West African cassava landraces (CMD2), and more recently, production of highly resistant cultivars carrying a quantitative trait loci (CMD3) [6–9]. Screening cassava germplasm for resistance to CMD traditionally involves cultivation under field conditions with exposure to transmission of CMGs mediated by the whitefly vector *Bemisia tabaci* for a growth cycle of 12–18 months [6, 10, 11]. Under contained conditions in the greenhouse or growth chamber, inoculation of cassava with CMGs can be achieved by a) graft inoculation from a CMG-infected host to healthy plants [12–14]; b) delivery of DNA genomes as infectious clones via microparticle bombardment [15–17]; c) A*grobacterium*-mediated inoculation of plants with cloned infectious DNA genomes [18]; or d) mechanical transmission of cloned viral DNA genomes by abrasion [19]. Irrespective of the inoculation method employed, CMD symptom development and severity is scored over a period lasting 12–22 weeks from the time of inoculation through the potential disease recovery process [15, 18]. During this time the resistant/tolerant cultivars are identified based on displayed recovery phenotype on newly formed leaves, while the susceptible cultivars remain symptomatic throughout.

Methods currently available for evaluating resistance and susceptibility to CMD in new cultivars, breeding lines or transgenic and gene edited events are therefore lengthy, space inefficient and require frequent assessment of leaf symptoms by skilled personnel. We report here the development of a simple screening system for determining resistance or susceptibility to CMD that can be completed within 2–4 weeks from the time of inoculation. This rapid screening system is based on virus-induced gene silencing (VIGS) of an endogenous *MeSPY* gene. The method described saves time and space in the greenhouse and enhances capability to allow screening of a large number of plants in a short period of time.

## Methods

### Construction of infectious VIGS clones

VIGS clones were generated from the virulent infectious clone EACMV-K201 described previously by Patil and Fauquet [20]. EACMV-K201 was produced from *East African cassava mosaic virus* (EACMV-KE[KE:Msa:K201:02]), DNA-A GenBank: AJ717541; and DNA-B GenBank: AJ704953 [21]. The EACMV-K201 DNA-A infectious clone was digested with *Hin* dIII/*Eco* RI (2082 bp) and *Eco* RI/*Bam* HI (1157 bp), and cloned into pBlueScript vector (Stratagene) as *Hin* dIII/*Bam* HI in a three-way ligation. The resulting construct was named p8200. p8200 was modified to introduce restriction sites *Nhe* I and *Avr* II near the 5′-region, and *Sbf* I near the 3′-region within the coding sequence of the coat protein (*CP*) gene using QuickChange Multi Site-Directed Mutagenesis Kit (Agilent Technologies, Inc.) with the primers listed in Table 2. The mutagenized infectious clone was confirmed by sequencing and named p8202.

The *Manihot esculenta* homolog of *A. thaliana SPINDLY* (*SPY*) gene (*MeSPY1* accession number Manes.09G052300.1) was cloned into p8202 at the CP site. A 452 bp fragment of *MeSPY1* (406–857 counted from start codon ATG) was amplified from plasmid p8103 that harbors the 2781 bp coding sequence of *MeSPY1* by introducing restriction sites *Nhe* I and *Sbf* I on the forward and reverse primer pairs, respectively (Table 2). The PCR product was cloned into Zero Blunt Topo (Invitrogen) first and positive clones digested and cloned into the *Nhe* I/*Sbf* I site of p8202 to generate the p8250 (hereafter named MeSPY1-VIGS). A non-target VIGS control was produced by amplification of 453 bp (235–687 bp counted from start codon ATG) from the erGFP [22] sequence of a binary vector erGFP-pCAMBIA2300 by introducing *Nhe* I and *Sbf* I sites on forward and reverse primer pairs, respectively, and cloned into p8202 to generate p8223 (GFP-VIGS). In order to generate a VIGS vector targeting cassava phytoene synthase (*MePSY2* gene, Manes.01G124200.1), a 452 bp fragment (350–801 bp counted from start codon ATG) was amplified introducing a *Sbf* I and *Nhe* I restriction site and cloned into the *Sbf* I and *Nhe* I site of 8202 to produce p8375 (MePSY2-VIGS).

### Cultivars and greenhouse growth conditions

Cassava cultivars used in this study are shown in Table 1 and included the CMD-susceptible cultivars 60444, Ebwanateraka and TME 7S; and CMD-resistant cultivars TME 3, TME 7 (Oko-iyawo), TME 204 and TMS 98/0505. Additional lines included CMD-susceptible plants [15] regenerated from friable embryogenic callus (FEC) of TME 204, TME 3 and TME 7 and CMD-resistant plants of cultivar TMS 98/0505 recovered from FEC following the method described by Chauhan*,* et al. [23]. All plants were micropropagated and established in the greenhouse as described previously [15, 24].

**Table 1:** Cassava cultivars and lines used for MeSPY1-VIGS, GFP-VIGS and MePSY2-VIGS challenge

Cultivar/line	Response to CMD	Remark
TME 204	Resistant	Recovers from CMD
TME 3	Resistant	Recovers from CMD
TME 7	Resistant	Recovers from CMD
TMS 98/0505	Resistant	Recovers from CMD
FEC- TMS 98/0505	Resistant	Recovers from CMD
60444	Susceptible	Does not recover from CMD
TME 7S	Susceptible	Does not recover from CMD
Ebwanateraka	Susceptible	Does not recover from CMD
FEC-TME 204	Susceptible	Does not recover from CMD
FEC-TME 3	Susceptible	Does not recover from CMD
FEC-TME 7	Susceptible	Does not recover from CMD

### Inoculation of VIGS clones and assessment of phenotype in the greenhouse

Four- to 6-week-old greenhouse-grown plants were inoculated with plasmid DNA of MeSPY1-VIGS, GFP-VIGS or MePSY2-VIGS vectors plus the DNA-B component of EACMV-K201 using a Helios® Gene Gun (BioRad, Hercules, California), following Beyene*,* et al. [15]. Approximately 75 ng each of the VIGS (DNA-A and DNA-B) components were used to inoculate each plant. Symptom and phenotype scoring after inoculation was performed in three manners. Plants challenged with GFP-VIGS were scored for development of CMD symptoms using an established visual scoring system with a scale of 0–5 [25]. Plants were scored starting 7–10 days post inoculation (DPI) and every 3–7 days thereafter for a total of 12 weeks [15]. For plants challenged with MePSY2-VIGS, visual assessment was made for presence or absence of chlorosis/bleaching. In the case of plants challenged with MeSPY1-VIGS, a new scoring system was employed by which plants were assessed for death of the shoot apical meristem (whole plant) starting 7–10 DPI and every 2–3 days thereafter for a maximum of 4 weeks. Data was expressed as incidence of plants showing shoot-tip necrosis/dead plants presented as the percent of the total number of plants inoculated at the end of the 4-week observation period.

### Nucleic acid extraction, qPCR and RT-qPCR

Plants were sampled for nucleic acid extraction by collecting leaves and shoot material 9 DPI with VIGS constructs. Tissues from three plants were pooled in a 50 mL Falcon tube and immediately flash frozen in liquid nitrogen. A total of four pooled samples collected from 12 plants were analyzed per treatment. Frozen samples were ground in a mortar and pestle to fine powder and freeze-dried overnight. Approximately 20–30 mg of freeze-dried tissue were used for nucleic acid extraction. Genomic DNA was extracted using DNeasy Plant Mini Kit (Qiagen, Hilden, Germany) and the resulting genomic DNA quantified and used for virus titer determination by qPCR and Southern blot analysis [15]. Total RNA was isolated using the Spectrum™ Plant Total RNA Kit, followed by on-column DNAse I treatment per manufacturer instructions (Sigma-Aldrich, St. Louis, MO). One microgram of total RNA was reverse transcribed using the PrimeScript™ RT reagent kit with gDNA Eraser (Takara Bio Inc., Japan). Expression levels of *MeSPY* in MeSPY1-VIGS-inoculated plants and control GFP-VIGS plants were quantified by RT-qPCR [26] using the primer pairs listed in Table 2, with approximately 10 ng reverse transcriptase template and SsoAdvanced™ Universal SYBER® Green Supermix (Bio-Rad Laboratories Inc.). The cassava *PP2A* gene was employed as an internal control for RT-qPCR [27].

**Table 2:** Primers used for in vitro mutagenesis, PCR, qPCR and RT-qPCR in this study

Primer number	Sequence	Target Gene (construct)	Purpose	Reference
823	CTTCCCAACTCTATGGGTGATGGCTAGCCTAGGAGTAACATCACTGACACATCG	EACMV-K201 (DNA-A)	Mutagenesis primer (introduces *Avr* II and *Nhe* I)	This study
824	CGATGTGTCAGTGATGTTACTCCTAGGCTAGCCATCACCCATAGAGTTGGGAAG			
825	ATAGATGCGTATTTTAAGCGTCGCACCTGCAGGATTTGAGGCATGTGTACATGC	EACMV-K201 (DNA-A)	Mutagenesis primer (introduces *Sbf* I)	This study
826	GCATGTACACATGCCTCAAATCCTGCAGGTGCGACGCTTAAAATACGCATCTAT			
857	GCTAGCCCAACACTTGTCACTACTTTC	erGFP	GFP-VIGS	This study
858	CCTGCAGGGAAAGGGCAGATTGTGTGGA			
907	GCTAGCGCTGAGTCATATCAGAAGGC	MeSPY1/Manes.09G052300.1	MeSPY1-VIGS	This study
908	CCTGCAGGATACCTTGATTGATGTCTCC			
1260	CCTGCAGGTAGACGTGAAACCAGATATTGTGC	MePSY2/Manes.01G124200.1	MePSY2-VIGS	This study
1261	GCTAGCAACACTCATTAATCCAACCGTCCC			
1066	CAAAGGTTAAATTGGAGGGAGACATC	MeSPY1/Manes.09G052300.1	RT-qPCR	This study
1067	GCATTTCACCATATGCAACACCAAGA			
833	TTGCAGAGGAAGATAGTGGGAATG	EACMV-K201 (DNA-A)	Probe for Southern blot	Beyene*,* et al. [15]
834	GAACGTGATGGGTTCCGCTG			
837	TGCAAGGCTCACACTTTCATC	MePP2A/Manes.09G039900.1	Reference gene for qPCR	Moreno*,* et al. [27]
838	CTGAGCGTAAAGCAGGGAAG			
1046	GGTCTTCCCTGTACGACTATC	EACMV-K201 (DNA-A)	qPCR (virus load)	Beyene*,* et al. [15]
1047	GGAACTTGAAGTCTGGGTTTCC			

## Results

### Production of EACMV-K201 VIGS clones and verification of infectivity

**Fig. 1:**
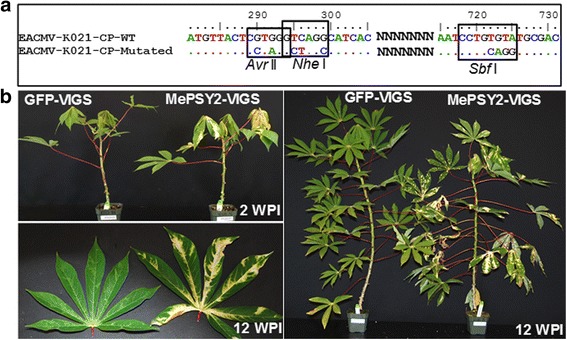
*East African Cassava mosaic virus* (EACMV-K201) infectious clone-based virus-induced gene silencing (VIGS) vector. **a** Coat protein (CP) nucleotide sequence of EACMV-K201 DNA-A showing position and mutated nucleotides that introduced *Avr* II and *Nhe* I near 5′-end and *Sbf* I near 3′-end. Nucleotide positions were counted from the start codon “ATG” of the CP. **b** Infectivity of the GFP-VIGS and MePSY2-VIGS targeting GFP and cassava phytoene synthase coding sequences, respectively, in wild-type cassava cultivar TME 7S. GFP-VIGS has no known target in the challenged cassava plant, and these plants display typical mosaic symptoms characteristic of CMD on leaves. MePSY2-VIGS-challenged plants show chlorosis on leaves. Pictures were collected at 2 and 12 weeks post inoculation (WPI)

A VIGS system was developed by modifying the CP nucleotide sequence of the DNA-A component of the virulent infectious clone EACMV-K201 [20] to generate the VIGS construct p8202. Mutations were designed to introduce restriction sites *Avr* II, *Nhe* I and *Sbf* I (Fig. 1a) in order to allow introduction of target sequences. The introduced mutations were confirmed by sequencing both strands of the modified CP sequences as shown (Fig. 1a) and further confirmed by restriction analysis (data not shown). To check infectivity of the p8202 VIGS vector, a 452 bp fragment from the coding sequence of the cassava carotenoid biosynthetic gene phytoene synthase (*MePSY2*) was cloned at the *Nhe* I/*Sbf* I site of p8202 to generate MePSY2-VIGS. In addition, a GFP-VIGS DNA-A component was generated by cloning a 453 bp sequence from erGFP [22] into the restriction sites generated within the CP sequence.

Five-week-old plants of the CMD-susceptible cassava line TME 7S were inoculated by Helios® Gene Gun microparticle bombardment of the modified DNA-A component plus the infectious clone of DNA-B. TME 7S is a CMD-susceptible version of TME 7 previously described by Kuria*,* et al. [28]. Plants inoculated with MePSY2-VIGS developed visible chlorosis/bleaching on the challenged leaves and then subsequently on systemic leaves within 10–15 days after bombardment (Fig. 1b). Bleaching of leaves persisted throughout the experimental period of 12 weeks (Fig. 1b). Plants challenged with GFP-VIGS (which does not have a target gene sequence within the cassava genome) showed typical but mild CMD symptoms on systemic leaves that persisted throughout the study period (Fig. 1b). Response of inoculated plants to MePSY2-VIGS and GFP-VIGS confirms that the VIGS constructs made by modifying EACMV-K201 are both infectious and efficacious in silencing gene expression in cassava.

### Silencing of *MeSPY* gene is lethal in CMD-susceptible cassava cultivars

Using the Arabidopsis *SPY* gene (AT3G11540.1) [29, 30] as a bait, two sequences, Manes.09G052300.1 (named *MeSPY1*) and Manes.08G028400.1 (named *MeSPY2*), were identified from the cassava v6.1 genome sequence [31]. The *MeSPY1* and *MeSPY2* sequences are 81% identical to Arabidopsis SPY at the amino acid level. Both MeSPY1 and MeSPY2 carry the conserved N-terminal tetratricopeptide repeat (TPR) domain, plus the novel serine and threonine *O*-linked N-acetylglucosamine transferase (OGT) [30] at the C-terminus of the protein (data not shown). The coding region of *MeSPY1* and *MeSPY2* genes are 91.80 and 90.78% identical to each other at the nucleotide and amino acid levels, respectively. The target sequence (452 bp) cloned into the VIGS vector from *MeSPY1* was obtained closer to the 5′-end, within the conserved TPR region. As *MeSPY1* and *MeSPY2* share high sequence identity (94.69%) at this selected region, both can be targeted for simultaneous silencing by MeSPY1-VIGS.

**Fig. 2:**
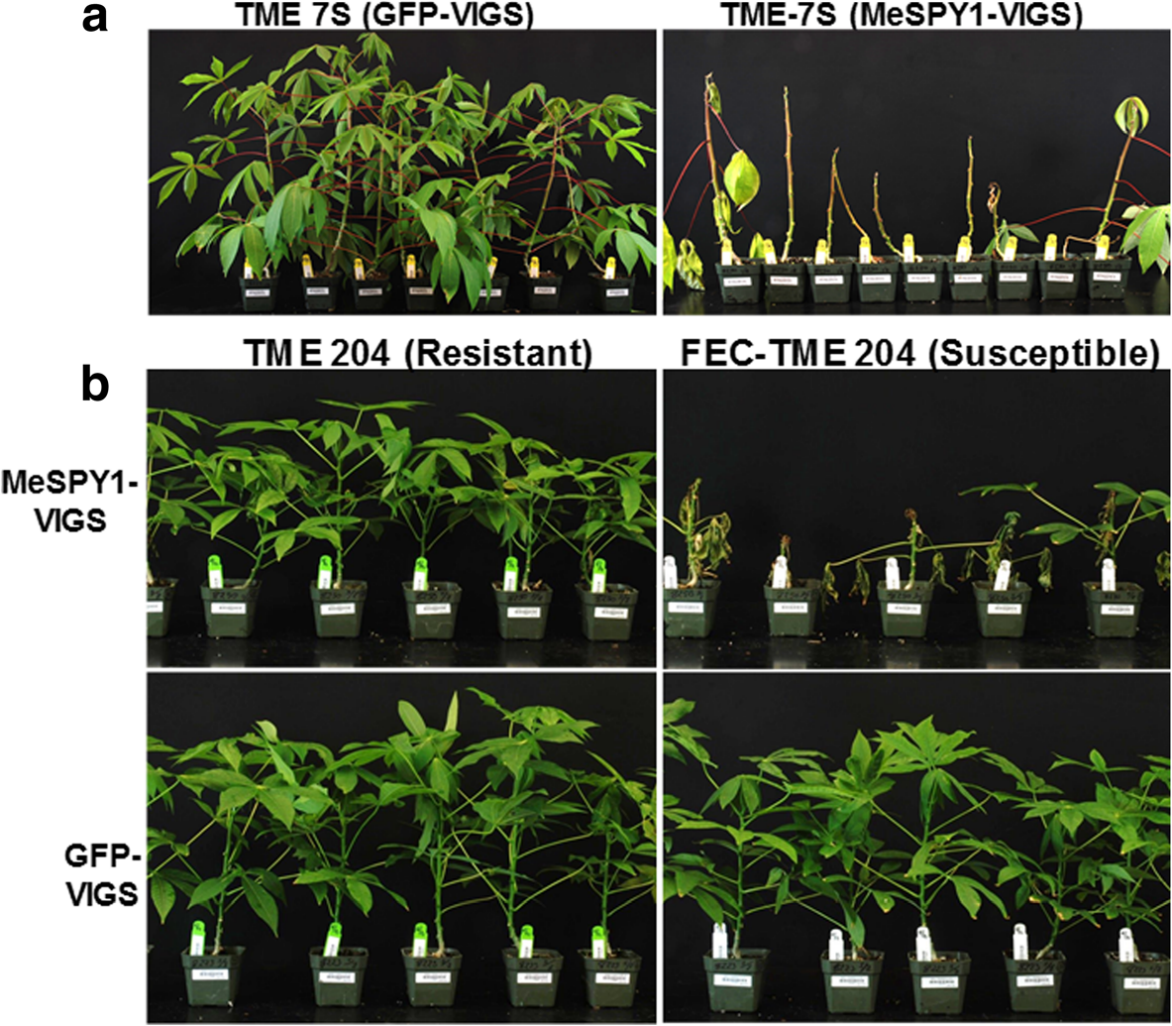
MeSPY1-VIGS-challenged susceptible and resistant cassava cultivars. **a** CMD-susceptible cassava cultivar TME 7S challenged with GFP-VIGS (left) and MeSPY1-VIGS (*right*) showing defoliation of leaves and death of shoot-tip at 31 days post-inoculation (DPI). **b** GFP-VIGS- (*bottom*) and MeSPY1-VIGS- (*top*) challenged wild-type TME 204 (resistant) and FEC-TME 204 (susceptible). Note symptoms of dead or dying plants in FEC-derived plants. Pictures captured at 24 DPI. All GFP-VIGS plants showed typical CMD symptoms on the leaves



The susceptible cassava cultivar TME 7S was challenged with MeSPY1-VIGS by Helios® microparticle bombardment. Inoculated plants did not show typical CMD symptoms but instead underwent wilting and withering of one or more leaves just above the inoculation site within 10–15 DPI. This was followed by shoot-tip necrosis within 12–21 DPI. Defoliation then progressed downwards to mature leaves below the inoculation site and along the main stem continuing until most of the plants had died within 2–4 weeks after inoculation with MeSPY1-VIGS (Fig. 2a). TME 7S plants challenged with the control GFP-VIGS showed typical CMD symptom development without shoot tip necrosis (Fig. 2a). In order to determine if this was a cultivar-specific response, the CMD2-type cultivar TME 204 (Table 1) was challenged with MeSPY1-VIGS and GFP-VIGS. Two types of TME 204 plants were used: wild-type TME 204, which shows development of CMD symptoms followed by recovery; and FEC-TME 204 lines. The latter TME 204 plants that were recovered through somatic embryogenesis lost their inherent resistance to CMD and showed no recovery from CMD after challenge with CMGs [15]. After challenge with MeSPY1-VIGS, all plants (10/10) of wild-type TME 204 displayed transient reduction in growth compared to the GFP-VIGS plants and then resumed normal growth without showing typical CMD symptoms. No shoot-tip necrosis was observed. In contrast, all FEC-derived TME 204 plants (8/8) displayed a similar phenotype to that described above for TME 7S, whereby elongation of the shoot was reduced, resulting in compact shoot-tips and wilting and withering of leaves just above the inoculation site. This was followed by death of the shoot-tip and wilting and defoliation of leaves that progressed downwards from the youngest to the oldest leaves. Time-lapse video of the process of shoot-tip necrosis and whole plant death in susceptible FEC-TME 204 and survival of the resistant wild-type TME 204 plant lines is presented (Additional file 1). All plants displayed shoot-tip necrosis with most plants dying within 2–4 weeks after challenge (Fig. 2b). Inoculation with GFP-VIGS resulted in plants of both the susceptible and resistant lines of TME 204 developing CMD symptoms. Wild-type TME 204 plants then underwent recovery from CMD to show no symptoms on newly formed leaves by 16 weeks after challenge. FEC-derived plants remained symptomatic throughout, in a manner consistent with previous observations [15]. This data indicates that silencing of MeSPY is lethal in the two susceptible cassava lines TME 7S and FEC-derived TME 204, but not in the resistant wild-type TME 204.

**Additional file 1:** Time-lapse video showing response of wild-type TME 204 (resistant to cassava mosaic disease, CMD) and FEC-TME 204 plants (susceptible to CMD) to inoculation with MeSPY1-VIGS. Both plant types were challenged with the modified EACMV-K201 (MeSPY1-VIGS) along with infectious DNA B clone. Images of challenged plants were captured every hour beginning from the 5^th^ day after inoculation for a total of 22 days using 5MP camera boards controlled by Raspberry Pi microcomputers. Images were then converted to a movie file using Apple iMovie. Only pictures collected every 2 hours and during daytime were presented. Note the susceptible FEC-TME 204 plants die while the wild-type TME 204 survive MeSPY1-VIGS challenge. (M4V 14035 kb)


**Fig. 3:**
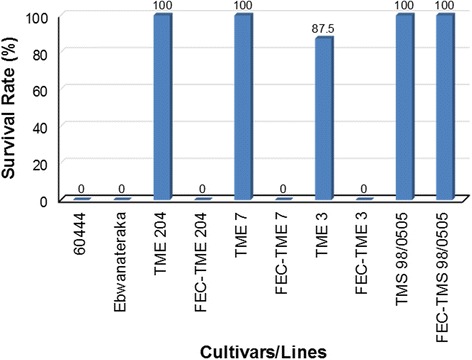
Survival rate of MeSPY1-VIGS-challenged cassava cultivars/lines. Four-week-old cassava seedlings were challenged with MeSPY1-VIGS and evaluated for survival at 4 weeks after challenge. Plants were considered dead if the shoot-tip had completely died and leaves had defoliated or were wilting and defoliating at evaluation period and are expressed as percent of challenged plants. Between 7 and 10 plants were challenged per cultivar/line and each experiment was repeated at least three times

**Fig. 4:**
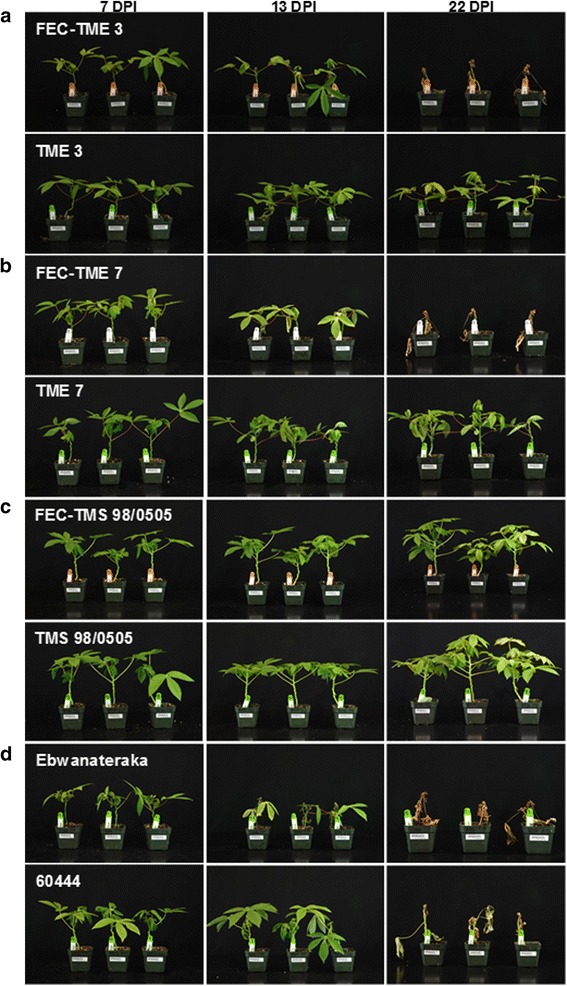
MeSPY1-VIGS-challenged cassava cultivars/lines. **a** FEC-derived and wild-type TME 3, **b** FEC-derived and wild-type TME 7, **c** FEC-derived and wild-type TMS 98/0505, **d** CMD-susceptible cassava cultivars Ebwanateraka and 60444. The FEC-derived lines and wild-type susceptible cultivars exhibit wilting of leaves at 13 DPI and complete necrosis of shoot-tip by 22 DPI. Pictures were collected at 7, 13 and 22 DPI

**Fig. 5:**
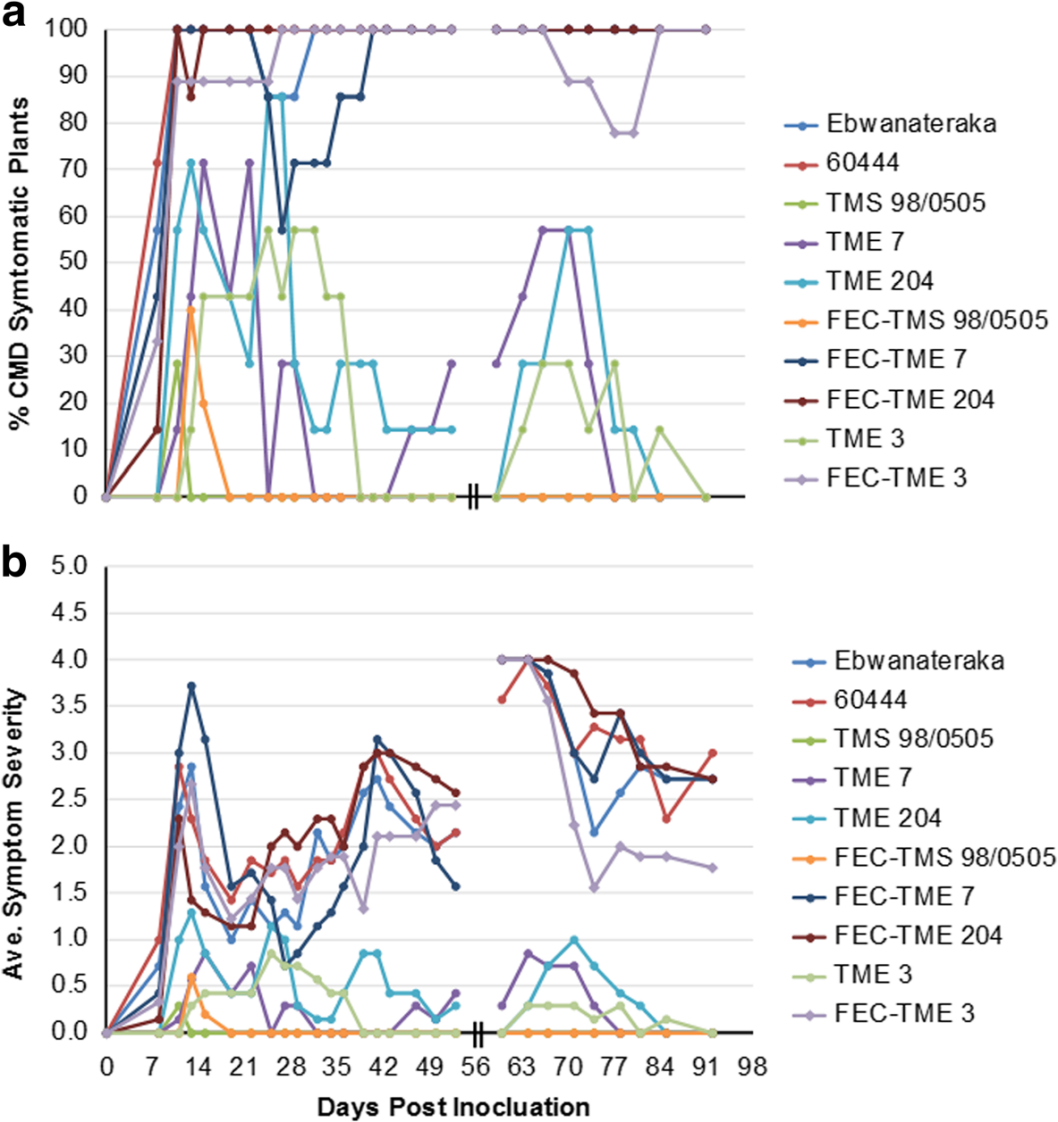
Response of cassava cultivars/lines to biolistic inoculation with GFP-VIGS under greenhouse conditions. **a** CMD incidence (**b**) average symptom severity scores (scale of 0–5). The resistant cassava cultivars showed full recovery from GFP-VIGS with 0 incidence and 0 average symptom severity at the end of the experiment (91 DPI), while the susceptible lines remained symptomatic with higher incidence (80–100%) and average severity score of 1.7–3.0 at the end of the experiment. Plant stems were cut back at 53 days after biolistic inoculation and CMD was assessed on new leaf growth. Breaks in the x-axis graph indicate lapse in shoot regrowth after this cut-back

MeSPY1-VIGS’s potential for use as a screening tool to determine resistance or susceptibility to CMD was investigated further by challenging additional cassava cultivars (Table 1). These included: a) CMD2-type resistant wild-type plants of cultivars TME 7 and TME 3, and CMD-susceptible plant lines derived from FEC of TME 7 and TME 3; b) CMD3-type resistant cultivar TMS 98/0505 (wild-type) and FEC-derived TMS 98/0505; and c) CMD-susceptible cassava cultivars 60444 and Ebwanateraka. Inoculation of these cassava accessions with MeSPY1-VIGS showed that CMD-susceptible germplasm (FEC-TME 3, FEC-TME 7, 60444 and Ebwanateraka) underwent wilting of leaves above the inoculation site and necrosis of shoot-tips within 10–21 DPI. This was followed by defoliation, culminating in death of the majority of plants within 4 weeks after inoculation (Figs. 3 and 4). The CMD-resistant wild-type cultivars TME 3, TME 7 and TMS 98/0505 survived challenge with MeSPY1-VIGS and displayed similar phenotype described above for wild-type TME 204 (Figs. 3 and 4). Plants of FEC-derived TMS 98/0505 behaved in a manner identical to wild-type TMS 98/0505 confirming their known resistance to CMD [15]. Likewise, wild-type and FEC-derived plants of CMD1-type cultivars NASE 3 (TMS 30572) and NASE 14 also survive MeSPY-VIGS (data not shown). All plant lines were also challenged with GFP-VIGS. Data collected for development of and recovery from CMD symptoms correlated with that from plants inoculated with MeSPY1-VIGS such that all plant lines that resisted challenge with MeSPY1-VIGS also showed recovery from CMD symptoms after inoculation with GFP-VIGS (Fig. 5).

### Suppression of *MeSPY* and virus titer in challenged plants

**Fig. 6:**
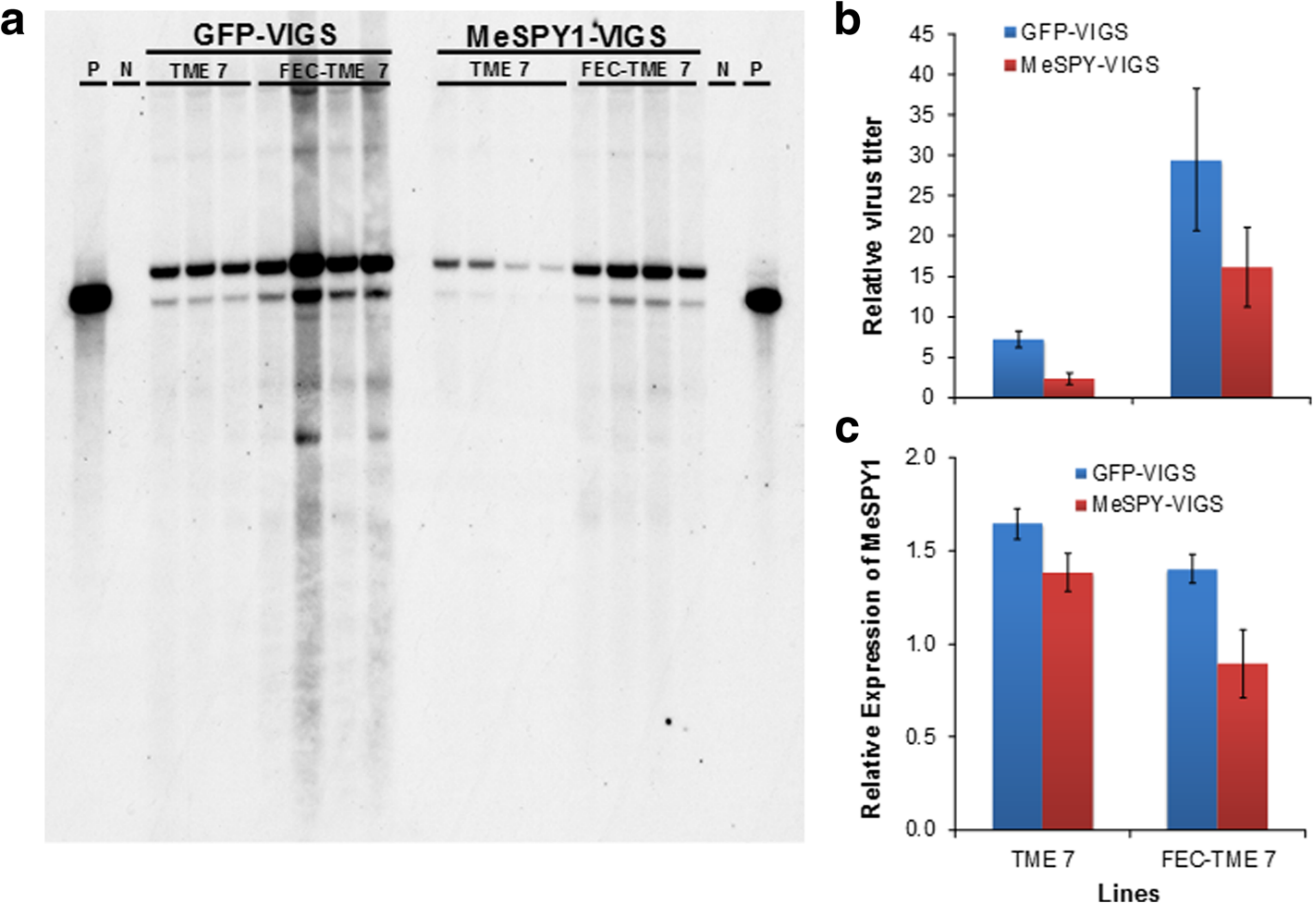
Virus titer in wild-type and FEC-derived TME 7 (Oko-iyawo) plants challenged with GFP-VIGS and MeSPY1-VIGS at 9 DPI. **a** Virus titer determination by Southern blot and **b** qPCR performed on total DNA extracted from leaves of wild-type (resistant to CMD) and FEC-derived (susceptible to CMD) plants. **c** RT-qPCR expression analysis of MeSPY. Higher virus titer is detected in both GFP-VIGS and MeSPY1-VIGS-challenged FEC-TME7 (susceptible plants) compared to the wild-type TME 7. Primers used for labelling probes for Southern blotting and for qPCR and RT-qPCR assays are shown in Table 1. Bars show SE (*n* = 4). P, positive control, N genomic DNA from unchallenged plants. Samples from three infected plants were pooled to make one sample, and a total of 4 samples (from 12 plants) were used per treatment combination

Expression of *MeSPY* after inoculation with MeSPY1-VIGS was quantified by RT-qPCR in the CMD-resistant wild-type cultivar TME 7 and susceptible FEC-TME 7 plants at 9 DPI. At this time, plants challenged with the control GFP-VIGS showed systemic CMD symptoms on young leaves above the inoculation site, while the MeSPY1-VIGS leaves were smaller and shoot-tips were compact relative to the GFP-VIGS. Relative expression of the *MeSPY* gene was found to be significantly reduced (33%; *p < 0.05*) in FEC-derived TME 7 plants challenged with MeSPY1-VIGS when compared to TME 7 wild-type plants and to the corresponding GFP-VIGS controls (Fig. 5c). Virus DNA titer was determined by qPCR (Fig. 5b) and Southern blot analysis (Fig. 5a) from the same samples collected at 9 DPI. TME 7 wild-type plants challenged with MeSPY1-VIGS and GFP-VIGS had significantly lower relative virus loads (2.36-fold and 7.23-fold, respectively) compared to the FEC-derived CMD-susceptible TME 7 plants (16.08 and 29.39, respectively). Greater virus DNA was detected in GFP-VIGS inoculated lines than MeSPY-VIGS inoculated lines (Fig. 6a). qPCR virus titer data was seen to be consistent with Southern blot data showing relatively more virus titer in the susceptible FEC-derived TME 7 compared to the wild-type TME 7.

## Discussion

Virus-Induced Gene Silencing (VIGS) has been used both in model and non-model plant systems to elucidate gene function [16, 32–35]. In cassava, a VIGS system was first reported based on an isolate of *African cassava mosaic Cameroon virus* (ACMV-CM) [16]. It was reported previously that this ACMV-CM infectious clone is less virulent than EACMV-K201 such that the CMD2-type cultivars TME 204, TME 3 and TME 7 (Oko-iyawo) are infected at low frequencies (0–30%) and develop only mild disease symptoms [15, 28]. This is less than ideal if robust suppression of gene expression is desired within an experimental system. The *East African cassava mosaic virus* isolate EACMV-K201 [21] is highly virulent and has been shown to infect all cassava genotypes [15, 28]. A new VIGS system was developed, therefore, based on EACMV-K201 by cloning target sequences into the coding region of the CP gene of the DNA-A component. Efficacy of this VIGS vector was confirmed by silencing the endogenous *MePSY2* gene in the CMD-susceptible cultivar TME 7S, resulting in visually detectable bleaching and production of chlorotic tissues throughout a 12-week observation period (Fig. 1b).

Screening cassava germplasm for resistance to CMD under field conditions requires many months [10, 36]. Under laboratory or greenhouse conditions this evaluation period is shorter but still needs 12–22 weeks [15, 18] to allow for full disease establishment and expression of the recovery phenotype typical for most cassava cultivars. Data presented here shows that the MeSPY1-VIGS system can be used as a quick screening tool to determine resistance and susceptibility to CMD (Figs. 2, 3 and 4). This was achieved by targeting the cassava *SPY* gene using a newly developed EACMV-K201-based VIGS vector delivered by biolistic inoculation. The established CMD scoring system for cassava involves visual assessment of symptoms based on a scale of 0–5 [25]. Experienced personnel are required to capture accurate data due to subtle presentation of disease symptoms in some cultivars. The symptom scores are recorded for each individual plant in an experiment often at a frequency of 1–2 times a week for 12–22 weeks depending on the cultivar, virus species and isolate used. Using the MeSPY1-VIGS screening system, only shoot-tip necrosis/death or whole plant death needs be scored, and only once or twice up to 4 weeks post inoculation. On average, this saves 8–18 weeks per inoculation experiment allowing 3–5 times more plants to be tested in the same time period and the same available greenhouse space. This MeSPY-VIGS screening system has been developed using known CMD-resistant and CMD-susceptible cassava cultivars and plant lines that have been well characterized under field and greenhouse conditions [8, 11, 15, 28]. Data generated (Figs. 1, 2, 3 and 4) corroborates accurately with the known CMD response of these cultivars, as further validated with challenge experiment using GFP-VIGS (Fig. 5), showing the robustness of the screening system.

The cause of shoot-tip necrosis that eventually leads to whole plant death in CMD-susceptible cassava lines is not clear. Both virus DNA and *MeSPY* transcript quantification showed significant differences between the CMD-resistant and CMD-susceptible plant lines, with CMD-susceptible plant lines having greater virus load than resistant lines (Fig. 6a and b) in a manner consistent with our previous report [15]. This viral load corresponded with an expected and significant reduction in *MeSPY* transcript in CMD-susceptible plants (Fig. 6c). *SPY* is involved in diverse physiological and developmental roles, including suppression of GA signaling [30], promotion of cytokinin response [37, 38] and enhancement of sensitivity to drought and salinity stress [39]. A *SPY* mutation in Arabidopsis caused elongation of the stem that phenocopies wild-type plants treated with GA [29, 30]. In Arabidopsis, *SPY* and *SECRET AGENT* (SEC) [40, 41] are the only proteins known to have *O*-linked N-acetylglucosamine transferase activity involved in posttranslational modification of other proteins. We tested if silencing of cassava *SEC* (*MeSEC*) mimics what has been observed in plants challenged with MeSPY1-VIGS as shown in this study (Figs. 1, 2, 3 and 4). Silencing of the cassava ortholog of Arabidopsis *SEC* did not cause lethality or any different phenotype in susceptible or resistant TME 204 and TME 7 cassava cultivars as compared to GFP-VIGS (data not shown), suggesting that *MeSPY* and *MeSEC* may not be functionally redundant. The cause of death after challenge with MeSPY1-VIGSs might therefore be due to the crucial role *MeSPY* plays in plant function and/or due to an unknown interaction with the geminiviruses. Transgenic plants overexpressing *MeSPY* and RNAi cassava lines are being recovered to further elucidate a putative role of *SPY* in CMD resistance.

The ability to discriminate between CMD-resistant and-susceptible lines using a simple MeSPY1-VIGS challenge has significant practical application. We recently reported that the CMD2-type cultivars TME3, TME 7 and TME 204 lose inherent resistance to CMD after passage through the process of somatic embryogenesis, and that this phenomenon occurs at an early stage after culture of an explant on auxin-containing media [31]. Meristem tip culture for virus elimination in infected cassava plants to allow movement of plants between countries or within a country, in vitro germplasm preservation and production of transgenic and gene edited plants often employ tissue culture procedures [42, 43]. The rapid CMD screening system developed in this study can easily be applied for determining preservation of CMD resistance after such tissue culture manipulations. Furthermore, the technique can be applied for high throughput screening of large numbers of progenies in a breeding pipeline under controlled greenhouse conditions. Besides understanding the molecular mechanisms involved in the phenotype observed in susceptible cassava lines reported here, investigation into the utility of SPY-VIGS as a screening tool in other plant-virus interactions is needed.

## Conclusion

A rapid and reliable screening method for determining resistance to CMD, one of the most important cassava viral diseases, has been developed. This screening system utilizes an EACMV-K201-based VIGS system to target the *MeSPY* gene and has proven effective across diverse cassava cultivars and lines. With this screening system in place, 3–5 times more plant lines can be screened with the same resources compared to previously available methods. It is therefore well suited for application for high throughput screening of breeding lines and transgenic and gene edited lines under controlled growth conditions.

## Abbreviations

ACMV-CM: *African cassava mosaic Cameroon virus*
CBSD: Cassava brown streak disease
CMD: Cassava mosaic disease
CMG: Cassava mosaic geminivirus
CP: Coat protein
DPI: Days post inoculation
EACMV: *East African cassava mosaic virus*
FEC: Friable embryogenic callus
*MePSY2*: *Manihot esculenta* phytoene synthase 2
*MeSEC*: *Manihot esculenta SECRET AGENT*
*MeSPY*: *Manihot esculenta SPY*
*SEC*: *SECRET AGENT*
*SPY*: *SPINDLY*
TPR: Tetratricopeptide repeat
VIGS: Virus-induced gene silencing
WPI: Weeks post inoculation
